# Supplementary material for: The temporospatial epidemiology of rheumatic heart disease in Far North Queensland, tropical Australia 1997–2017; impact of socioeconomic status on disease burden, severity and access to care
Source: PLoS Negl Trop Dis. 2021 Jan 14;15(1):e0008990. doi: 10.1371/journal.pntd.0008990 (PMC7840049; doi:10.1371/journal.pntd.0008990)
Supplement: S1 STROBE Checklist — (DOCX) [file pntd.0008990.s001.docx]

STROBE Statement—Checklist of items that should be included in reports of ***cohort studies***

|  | Item No | Recommendation |
| --- | --- | --- |
| **Title and abstract** | 1 | (*a*) Indicate the study’s design with a commonly used term in the title or the abstract. **This is provided in line 37.** |
|  |  | (*b*) Provide in the abstract an informative and balanced summary of what was done and what was found. **This is provided in lines 42-61.** |
| Introduction | | |
| Background/rationale | 2 | Explain the scientific background and rationale for the investigation being reported. **This is provided in lines 101-142.** |
| Objectives | 3 | State specific objectives, including any prespecified hypotheses. **This is provided in lines 143-149.** |
| Methods | | |
| Study design | 4 | Present key elements of study design early in the paper. **This is provided in lines 151-162.** |
| Setting | 5 | Describe the setting, locations, and relevant dates, including periods of recruitment, exposure, follow-up, and data collection. **This is provided in lines 151-180.** |
| Participants | 6 | (*a*) Give the eligibility criteria, and the sources and methods of selection of participants. Describe methods of follow-up. **This is provided in lines 153-180.** |
|  |  | (*b*) For matched studies, give matching criteria and number of exposed and unexposed. **Not applicable** |
| Variables | 7 | Clearly define all outcomes, exposures, predictors, potential confounders, and effect modifiers. Give diagnostic criteria, if applicable. **This is provided in lines 153-180.** |
| Data sources/ measurement | 8* | For each variable of interest, give sources of data and details of methods of assessment (measurement). Describe comparability of assessment methods if there is more than one group. **This is provided in lines 153-180.** |
| Bias | 9 | Describe any efforts to address potential sources of bias. **Not applicable** |
| Study size | 10 | Explain how the study size was arrived at. **This is provided in lines 153-159.** |
| Quantitative variables | 11 | Explain how quantitative variables were handled in the analyses. If applicable, describe which groupings were chosen and why. **This is provided in lines 156-179.** |
| Statistical methods | 12 | (*a*) Describe all statistical methods, including those used to control for confounding **This is provided in lines 183-190.** |
|  |  | (*b*) Describe any methods used to examine subgroups and interactions **This is provided in lines 183-189.** |
|  |  | (*c*) Explain how missing data were addressed. **This is provided in lines 189-190** |
|  |  | (*d*) If applicable, explain how loss to follow-up was addressed. **This is provided in lines 189-190** |
|  |  | (*e*) Describe any sensitivity analyses. **Not applicable** |
| Results | | |
| Participants | 13* | (a) Report numbers of individuals at each stage of study—eg numbers potentially eligible, examined for eligibility, confirmed eligible, included in the study, completing follow-up, and analysed. **This is provided in lines 191-197.** |
|  |  | (b) Give reasons for non-participation at each stage. **Not applicable** |
|  |  | (c) Consider use of a flow diagram. **Not applicable** |
| Descriptive data | 14* | (a) Give characteristics of study participants (eg demographic, clinical, social) and information on exposures and potential confounders. **This is provided in lines 199-205.** |
|  |  | (b) Indicate number of participants with missing data for each variable of interest. **This is provided in lines 287-289 and 340-341.** |
|  |  | (c) Summarise follow-up time (eg, average and total amount). **This is provided in lines 280-281** |
| Outcome data | 15* | Report numbers of outcome events or summary measures over time. **This is provided in lines 298-345 and figures 1,2,3,6 and 7.** |
| Main results | 16 | (*a*) Give unadjusted estimates and, if applicable, confounder-adjusted estimates and their precision (eg, 95% confidence interval). Make clear which confounders were adjusted for and why they were included. **This is provided in lines 257-345.** |
|  |  | (*b*) Report category boundaries when continuous variables were categorized. **This is provided in table 3.** |
|  |  | (*c*) If relevant, consider translating estimates of relative risk into absolute risk for a meaningful time period. **Not applicable** |
| Other analyses | 17 | Report other analyses done—eg analyses of subgroups and interactions, and sensitivity analyses. **This is provided in lines 263-266, 321-324 and 332-334** |
| Discussion | | |
| Key results | 18 | Summarise key results with reference to study objectives. **This is provided in lines 356-362.** |
| Limitations | 19 | Discuss limitations of the study, taking into account sources of potential bias or imprecision. Discuss both direction and magnitude of any potential bias. **This is provided in lines 457-475.** |
| Interpretation | 20 | Give a cautious overall interpretation of results considering objectives, limitations, multiplicity of analyses, results from similar studies, and other relevant evidence. **This is provided in lines 409-447.** |
| Generalisability | 21 | Discuss the generalisability (external validity) of the study results. **Not applicable** |
| Other information | | |
| Funding | 22 | Give the source of funding and the role of the funders for the present study and, if applicable, for the original study on which the present article is based. **Not applicable** |
