# Supplementary material for: The temporospatial epidemiology of rheumatic heart disease in Far North Queensland, tropical Australia 1997–2017; impact of socioeconomic status on disease burden, severity and access to care
Source: PLoS Negl Trop Dis. 2021 Jan 14;15(1):e0008990. doi: 10.1371/journal.pntd.0008990 (PMC7840049; doi:10.1371/journal.pntd.0008990)
Supplement: Attachment — Submitted filename: plos pathogen review.docx [file pntd.0008990.s002.docx]

This is an interesting study. The authors show that the prevalence and incidence of acute rheumatic fever (ARF) and rheumatic heart disease (RHD) are still high in the Far North Queensland (FNQ) Indigenous population of Australia.

Comments:

1. In this retrospective study, the authors analysed the patient data in the RHD registry in FNQ over 20 years between 1997-2017. However, RHD wasn’t a notifiable disease in Australia until 2018. While this weakness of the study-design was mentioned in the manuscript, and possibly underestimates the disease burden in FNQ, we do not know the extent of the underestimation. Do the authors know what proportion of health centres/clinicians in FNQ routinely maintained the RHD registry, and if so could the authors estimate the extent of possible underestimation of the disease?
2. Based on the data in Figure 1, the authors state that the incidence of ARF remained the same during study period. Please clarify that the number of cases each year are indeed new cases for each year. Please state how ARF was diagnosed and whether the criteria used remained the same for all communities throughout the study period.
3. Can the authors give reasons why the incidence of RHD increased in the study period despite the incidence of ARF remain the same? Has there been proactive prophylactic regimen for ARF patients during this period in the FNQ?
4. The authors state that complete secondary prophylaxis data was available only from 2007. Does it mean that the first 10 years of this study prophylaxis data may not be accurate? Please comment.
5. How was the disease severity determined? Please add this to the methods section.
6. The data shows that the Indigenous patients living in rural and remote locations or in lower socioeconomic conditions were less likely to have severe RHD than people living in urban areas or of higher socioeconomic conditions. This is an interesting and important point for health care providers. Indeed the results are quite counter-intuitive. It is important that the authors provide possible reasons for this observation.
