# Supplementary material for: The temporospatial epidemiology of rheumatic heart disease in Far North Queensland, tropical Australia 1997–2017; impact of socioeconomic status on disease burden, severity and access to care
Source: PLoS Negl Trop Dis. 2021 Jan 14;15(1):e0008990. doi: 10.1371/journal.pntd.0008990 (PMC7840049; doi:10.1371/journal.pntd.0008990)
Supplement: Attachment — Submitted filename: PLOS NTD Response to reviewers.docx [file pntd.0008990.s003.docx]

Dear Professor Vinetz and Dr Fischer,

Thank you for the opportunity to submit a revised version of our manuscript and to address the concerns of the reviewers. Please find below our point by point responses to their comments. The Word document is colour-coded (reviewer queries in blue, our responses in black) to facilitate legibility. As reviewer 1 uploaded a separate file, we have responded to his/her comments at the end of the document.

Methods
-Are the objectives of the study clearly articulated with a clear testable hypothesis stated?
-Is the study design appropriate to address the stated objectives?
-Is the population clearly described and appropriate for the hypothesis being tested?
-Is the sample size sufficient to ensure adequate power to address the hypothesis being tested?
-Were correct statistical analysis used to support conclusions?
-Are there concerns about ethical or regulatory requirements being met?

Reviewer #1: Attached.

**Response:** See response to all of reviewer 1 comments – which were attached as a separate file - at the end of this document.

Reviewer #2: no comments

Reviewer #3: Methods seem appropriate to the objectives of the study. It is anticipated that the Australian Indigenous population residing in the remote areas will have other health conditions additional to RHD. It would be of interest to consider other relevant diseases/conditions from patients health records.
**Response**: The reviewer is absolutely correct to note that many of the patients in this study were also suffering from a number of comorbidities. Macrovascular disease, cancer, sepsis and complications of the metabolic syndrome - to name just a few – are all more common in the local Indigenous population. We have expanded our documentation of the causes of death in the Indigenous population to highlight this (lines 341-345). We have also expanded the discussion to highlight that there are many communicable and non-communicable diseases that are also seen more frequently in Indigenous Australians, particularly those living in remote socioeconomically disadvantaged locations (lines 380-382). Detailed description of the all the health conditions whose burden is disproportionately borne by the Indigenous population is probably beyond the scope of this article which has a focus on RHD. However, we do cite the 2018 report of the Chief Health Officer of Queensland (reference 33) - with the URL where the full text can be accessed - for the interested reader to examine the relationship between Indigenous status and the incidence of a variety of health conditions – other than RHD - in the FNQ region.

Reviewer #4: (No Response)
--------------------

Results
-Does the analysis presented match the analysis plan?
-Are the results clearly and completely presented?
-Are the figures (Tables, Images) of sufficient quality for clarity?

Reviewer #1: attached
**Response**: See response to all of reviewer 1 comments – which were attached as a separate file - at the end of this document.
Reviewer #2: There is a decline in surgeries 2016-2017 that is quite significant compared to the upward moving trend from 1997 - 2015. Author should address why this may be the case - or at least discuss.  Statement is true that number of surgeries increased WITHIN the study period (97-15) but not OVER the entire study period (97-17).
**Response**: Temporal trends in this manuscript were determined using an extension of the Wilcoxon rank-sum test, which was developed by Cuzick (Cuzick, J. 1985. A Wilcoxon-type test for trend. Statistics in Medicine 4: 87-90). This is a very standard - and commonly used - statistical method for examining trends over time. It is not necessary for the absolute number of events to increase every single year for there to be a statistically significant increase in the event over a defined period of time. It is rather the *trend* in their incidence which is examined. We have expanded our description of our statistical methodology in the methods section to allow the reader to understand our approach.

Reviewer #3: Should the numbers of ARF diagnosis (Fig 1) be presented in the same scale as RHD cases (Fig 2)? It is difficult to compare the two values otherwise to see the correlation.

**Response**: While ARF and RHD are part of the same disease process, it is not the case that every patient with confirmed ARF develops RHD. Furthermore, the RHD may not become apparent – even on echocardiogram – until several years have elapsed. It is also certainly the case that many adults who have RHD diagnosed do not recall an episode of ARF (as was the case in almost half of the patients in this cohort (lines 212-213).

Moreover, while RHD can affect children and adults, the risk of ARF declines over time. While adults can certainly develop ARF, it is classically a disease of childhood and early adulthood. Therefore, the populations at risk are different, affecting the denominator that we might use to define the incidence. In the case of RHD this is effectively the entire population. In the case of ARF this is really – to a first approximation – those less than 30 years of age.

What we are trying to show in figure 1 is that despite Australia’s status as a wealthy country with a universal health care system, cases of ARF are still being diagnosed. It could be argued that *any* number of cases is important to highlight.

Although Figure 2 does show the incidence - new cases/100,000/year) providing the reader with an understanding of the burden of disease - it is likely that the majority of new RHD diagnoses were actually the result of an episode – or episodes – of ARF many years previously. This is emphasised by the fact that the median (IQR) age at RHD diagnosis was 29 (17-44) years, much later than the age that ARF typically occurs. The inciting ARF – that was responsible for the RHD - was either not recognised at the time it occurred (it was mild or not clinically suspected) or the individual was not able to access the echocardiography that is required to establish the RHD diagnosis concurrently. This is a point that we highlight in the discussion (lines 412-416 and lines 423-430)

Under Mortality: It is stated that although more numbers of non-indigenous patients died (14%) during the study period than indigenous patients (5%), indigenous patients died at a younger age. Only 38% of death was directly linked to RHD, 43% was not RHD-related, and 18% had no medical record.

Could the authors clarify how many of RHD related death (e.g. out of 38%) were indigenous patients? It is possible that the indigenous patients died at a younger age due to health conditions/factors other than RHD while non-indigenous patients died from RHD? If that is the case, abstract should be amended to keep the indigenous age factor with death due to RHD in context.

**Response**: This is an excellent suggestion. We have added this information to the results (lines 341-345) which we agree shows the burden of RHD-related death in younger Indigenous patients. We have also amended the abstract (lines 54-56) to highlight this point.

Reviewer #4: (No **Response**)
--------------------

Conclusions
-Are the conclusions supported by the data presented?
-Are the limitations of analysis clearly described?
-Do the authors discuss how these data can be helpful to advance our understanding of the topic under study?
-Is public health relevance addressed?

Reviewer #1: attached
**Response**: See Response to all of reviewer 1 comments – which were attached as a separate file - at the end of this document.

Reviewer #2: no comments

Reviewer #3: The authors discussed in details about the impact of overcrowding in the remote indigenous communities. Risks for infection GAS, ARF, RHD and other respiratory, gastrointestinal, eye and ear diseases. Scabies is a highly prevalent skin disease among remote indigenous communities in North Queensland. It would be appropriate to discuss scabies as possible risk factor for GAS infections and secondary complications such as ARF and RHD.

**Response**: We agree with the reviewer. We have modified the discussion to highlight the role that scabies may play (lines 396-397).

Line 409: Please include explanation for the significance of dental review. Is dental review representative of access to health care in these communities?

**Response**: In lines 437-438 and lines 470-474 we do discuss aspects of the dental review which are important to reduce the risk of infective endocarditis in people living with RHD. We highlight that the data surrounding dental service may be incomplete, but we also highlight that the provision of dental services is almost certainly inadequate presently.

However, this is not necessarily representative of access to all health care in these communities. Access to different components of health care is not uniform in FNQ, this is particularly the case with specialist services. Although there is limited access to public dental care, almost 94% (643/686) of the cohort had received specialist medical care and the patents had received a median (IQR) of 5 (3-8) echocardiograms over the course of the study. Conversely – as we highlight – patients living in rural and remote locations (with a lower SEIFA score) were less likely to receive valve surgery.

The proportion of patients accessing dental care, receiving specialist medical review and cardiac surgery are all presented, stratified by Indigenous status (Table 1), Remote residence (table 2) and socioeconomic disadvantage (table 3). Access to care (lines 380-386) and ways that we might improve this are also discussed (lines 441-447 and 453-456).

Line 442: limitation in using ICD coding to define patient’s hospitalisation was discussed. Please provide a brief explanation of what ICD coding is and why this is a limitation.

**Response**: Expansion of this point may be beyond the scope of the article which focuses on the public health approached to improve the care of patients living with RHD in the region. However, there are two references for the interested reader that examine this issue (references 52 and 53). Fundamentally, ICD coding relies on clinical documentation and the coders’ interpretation of this documentation. If there are errors in either, then this will introduce errors into the ICD coding. Detailed discussion of this issue, we believe, may distract the reader from the main points of the article, however we are happy to expand the discussion around the issue if the Editor feels that it would enhance the manuscript.

Reviewer #4: (No **Response**)
--------------------

Editorial and Data Presentation Modifications?
Use this section for editorial suggestions as well as relatively minor modifications of existing data that would enhance clarity. If the only modifications needed are minor and/or editorial, you may wish to recommend “Minor Revision” or “Accept”.

Reviewer #1: (No **Response**)

Reviewer #2: Two minor grammar errors line

- line 3 of the Introduction:
Improvements in the standard of living and the development of more effective treatment of group A streptococcal (GAS) infections in (remove in) during the 20th century

- line 1 of Comparison of Aboriginal and Torres Strait Islander Australians - change islander to Islander (capital I).

**Response**: We thank the reviewer for highlighting these typographical errors. We have amended the text accordingly.

Reviewer #3: Accept.

Reviewer #4: (No **Response**)
--------------------
Summary and General Comments
Use  this section to provide overall comments, discuss strengths/weaknesses of the study, novelty, significance, general execution and scholarship. You may also include additional comments for the author, including concerns about dual publication, research ethics, or publication ethics. If requesting major revision, please articulate the new experiments that are needed.

Reviewer #1: (No **Response**)

Reviewer #2: Excellent paper, well written and concise. Will add new knowledge to the subject area and provide advocacy for change.

Would recommend formally acknowledging the contribution of the RHD Register and Control Program in an Acknowledgements section rather than in the methods section. Alternatively, if the significant contribution was from individual(s) could consider offering an authorship. This is becoming an appropriate/respectful way of recognising the contribution of community workers/organisations.

**Response**: In fact, two of the authors - Mellise Anderson and Erin Howell – are members of the RHD Control Program. In the initial submission, their affiliation was recorded as the Tropical Public Health Unit. However, in the revised manuscript we have also documented that they are members of the RHD Control Program to demonstrate their commitment to this project and the ongoing care of people living with RHD in the region, as well as acknowledging the RHD Control program’s contribution in a more formal manner.

In the introduction, elimination of malaria/filariasis/strongyloides/leprosy is mentioned. May want to consider discussing that in addition to RHD, scabies is still endemic in many regions where RHD occurs and efforts to address scabies are needed. Especially as scabies is considered to contribute to RHD development.

**Response**: We agree with the reviewer. As noted above, although scabies is seen far less frequently in FNQ than in the Northern Territory we have modified the discussion to highlight the role that scabies may play (lines 396-397).

Reviewer #3: Manuscript is well written. This is a retrospective analysis of prevalence or Rheumatic Heart Disease (RHD) in far North Queensland using RHD registry (1997-2017). Although high prevalence of RHD in the remote indigenous communities are well-known, it is confronting to see the dispropionately high rates of RHD in younger age group.

It would be interesting for the authors to speculate why RHD is highly prevalent in younger age group for indigenous community compared with non-indigenous population. Could the authors also explain why despite high prevalence of RHD, almost half of RHD indigenous cases are mild to moderate (48%, 27% respective) and only 24% severe.

**Response**: We agree that the results are confronting, however, we feel that they are – at least to some degree – the result of enhanced detection. Since 2011 a paediatric cardiologist has been employed and since 2014 there has been significant expansion of the adult specialist outreach service. The adult physicians - aware of the local burden of RHD and keen to avoid its evolution - will frequently also perform echocardiograms on children during their visits. Both services use Vivid-I portable ultrasounds which permits rapid echocardiography during the clinical consultation.

The greater number of specialist visits has lowered the threshold for echocardiography being performed. Accordingly, echocardiography has become more proactive rather than simply being employed in symptomatic individuals. There has also been a focus on young women who are – or plan to become – pregnant, in an effort to identify women at risk of RHD-related complications during their pregnancy. All these factors will tend to identify a greater number of young people with RHD and also less severe disease.

Discussion of these issues has been expanded in lines 412-416 and lines 422-430.

Reviewer #4: 1. Figure 1, 2 and 3 - To make the graph more informative, I would like to suggest to have new RHD admissions of the each year to be indicated in a different colour in the same bar of total RHD cases.

**Response**: As the figures are showing *incidence* (new cases/100,000/year) each bar does in fact show new cases. However, we have amended the figure titles to make it clearer that each bar represents new cases.

2. It would be good to discuss the potential reasons to have low RF specifically in the years with high RHD. In addition, if the increased health services have contributed to high RHD cases in the recent year, how authors would describe the low/steady case numbers of RF?

**Response**: While ARF and RHD are part of the same disease process, it is not the case that every patient with confirmed ARF develops RHD. They are also diagnosed in a different manner: ARF is diagnosed using clinical and laboratory criteria: cases can therefore be - and usually are - diagnosed by primary health workers. Meanwhile RHD requires echocardiography confirmation which is not performed by primary health workers in FNQ, it instead requires specialist assessment. Furthermore, the RHD may not become apparent – even on echocardiogram – until several years have elapsed.

ARF classically occurs in children and young adults (although it is certainly documented as occurring in older patients as well). The fact that the median (IQR) age at the time of RHD diagnosis in this cohort was 29 (17-44) years suggests that the ARF and RHD are not being diagnosed concurrently. The RHD is often being diagnosed when the patient is an adult and having an echocardiogram for another reason. Indeed only 358/686 (52.2%) of the RHD patients had a history of ARF (lines 212-213).

The recent expansion of the adult specialist outreach programs and the resulting lower threshold for echocardiography is likely to explain why the incidence of RHD appears to be rising significantly – particularly in the rural and remote settings that the services visit - despite the incidence of ARF remaining stable. The program expanded in 2014 and after this time there is a sharp increase in the incidence of new RHD diagnoses (figures 2 and 3)

**Response** to reviewer 1 comments – which were attached as a separate file:

This is an interesting study. The authors show that the prevalence and incidence of acute rheumatic fever (ARF) and rheumatic heart disease (RHD) are still high in the Far North Queensland (FNQ) Indigenous population of Australia.

Comments:

1. In this retrospective study, the authors analysed the patient data in the RHD registry in FNQ over 20 years between 1997-2017. However, RHD wasn’t a notifiable disease in Australia until 2018. While this weakness of the study-design was mentioned in the manuscript, and possibly underestimates the disease burden in FNQ, we do not know the extent of the underestimation. Do the authors know what proportion of health centres/clinicians in FNQ routinely maintained the RHD registry, and if so could the authors estimate the extent of possible underestimation of the disease?

**Response**: The reviewer makes a very good point highlighting the fact that RHD was not a notifiable disease until after the study period. We do however acknowledge this in the limitations section of the discussion.

In the absence of population screening there is little doubt that we are significantly underestimating the burden of RHD, although this is the case wherever RHD is seen, a New England Journal of Medicine study (reference 51) which was performed in Asia and Africa suggests that systemic community screening echocardiography will identify approximately 10 times the number of RHD cases as clinical-based screening.

However, the extent of under-estimation - in the absence of community screening - is difficult to determine. It is not the case that health centres or clinicians opted in - or out – of providing data to the RHD register before RHD was made a notifiable disease and so it is not possible to answer the reviewer’s query about the “proportion of health centres/clinicians in FNQ (that) routinely maintained the RHD registry.”

In Queensland, conditions with a laboratory-based diagnosis – for instance hepatitis A-E, HIV, arboviruses - are automatically notified to public health authorities. Protocols are in place for all public and private laboratories in the state to do this. However, neither ARF nor RHD are pathological diagnoses. They require the individual clinician to report the case - via telephone call or email - to the public health unit. This is an imperfect process.

While many local clinicians take this responsibility seriously, clinicians who are new to the region – and there is a relatively high turnover of staff in remote communities – may not notify the RHD Control Program. It is also the case that many clinicians who have been working in the region for years have still not received appropriate education about the notification process. Inevitably even among the clinicians who are aware of the RHD register there may be instances where they forget to make the notification.

However, if these patients subsequently have an echocardiogram at a public hospital in Queensland and if RHD is reported this information will be fed back to the Queensland Cardiac Outcomes Registry (QCOR), which then, in turn, passes it on the RHD register.

To address the point that the reviewer has raised we have expanded the discussion to highlight some of these issues (lines 457-464).

2. Based on the data in Figure 1, the authors state that the incidence of ARF remained the same during study period. Please clarify that the number of cases each year are indeed new cases for each year. Please state how ARF was diagnosed and whether the criteria used remained the same for all communities throughout the study period.

**Response**: The cases were indeed new cases; the title of the figure has been amended to highlight this. Cases of possible, probable or confirmed ARF were reported to the RHD register by health workers who used their clinical judgement. The diagnosis was not standardised. It is clinical practice to use the Jones’ criteria locally – which are published widely in local guidelines - although they evolved over the study period and there are well-described issues with their sensitivity and specificity. We have modified the methods section to make this clearer (lines 159-162)

3. Can the authors give reasons why the incidence of RHD increased in the study period despite the incidence of ARF remain the same? Has there been proactive prophylactic regimen for ARF patients during this period in the FNQ?

**Response**: Several of the reviewers have raised this issue which is, we agree, an important one to highlight. It is likely to be explained by the expanded adult and paediatric specialist outreach programs and the resulting lower threshold for echocardiography. This is probably why the incidence of RHD appears to be rising significantly despite the incidence of ARF remaining stable.

It is notable that the incidence of RHD climbs significantly in rural and remote locations after 2011 when the paediatric specialist outreach program commenced and further still after 2014 when the adult medicine outreach program was expanded (figure 3). This is now specifically highlighted in the discussion (lines 412-416).

ARF classically occurs in children and young adults (although it is certainly documented as occurring in older patients as well). The fact that the median (IQR) age at the time of RHD diagnosis was 29 (17-44) years suggests that the ARF and RHD are not being diagnosed concurrently. The RHD is being diagnosed when the patient is an adult and having an echocardiogram for another reason. Indeed only 358/686 (52.2%) of the RHD patients had a history of ARF (line 416).

4. The authors state that complete secondary prophylaxis data was available only from 2007. Does it mean that the first 10 years of this study prophylaxis data may not be accurate? Please comment.

**Response**: We thank the reviewer for highlighting this issue. We can see how the original phrasing “Complete secondary prophylaxis data were only available from 2007” was ambiguous. We have amended the text to highlight the fact that adherence to secondary prophylaxis prior to 2007 was not included in the analysis (lines 172-173).

5. How was the disease severity determined? Please add this to the methods section.

**Response**: RHD severity was determined using the echocardiogram reports of the specialist physician who performed and/or reported the study. The text has been amended to highlight this (lines 156-157).

6. The data shows that the Indigenous patients living in rural and remote locations or in lower socioeconomic conditions were less likely to have severe RHD than people living in urban areas or of higher socioeconomic conditions. This is an interesting and important point for health care providers. Indeed the results are quite counter-intuitive. It is important that the authors provide possible reasons for this observation.

**Response**: We agree with the reviewer that this finding seems, on first inspection, quite counter-intuitive. Its explanation is likely to be multifactorial.

The first point to make is that while there was a statistically significant difference (p=0.03) in the number of patients with severe RHD in rural and remote locations compared with urban settings (76/344 (22.1%) versus 101/342 (29.5%)), it is important to remember that with a cut-off of 0.05 for significance, 1 in 20 comparisons will – by chance – have a p value of < 0.05. Given the numerous comparisons that we have performed in the study, some statisticians might suggest that we choose a more stringent cut-off for statistical significance than p<0.05.

However, if we are to accept that the difference between the proportion of severe cases in the two locations is real, there are several explanations for this finding.

The first is that it may be linked to the enhanced recent service delivery in the disadvantaged rural and remote settings. As we report in table 2, patients in these locations were more likely to receive specialist review than those living in urban locations (333/344 (96.8%) versus 310/342 (90.6%), p=0.001), lowering the threshold for echocardiography, potentially identifying a greater number of cases of milder disease. If we examine the period after 2011 when outreach services to rural and remote settings expanded there is a trend to less severe disease being identified in rural and remote locations on the patient’s first echocardiogram, although this does not reach statistical significance.

**Findings on first echocardiogram after 2011:**

| Disease severity | Mild | Moderate | Severe | All |
| --- | --- | --- | --- | --- |
| Rural and remote location | 55 (60.4%) | 21 (23.1%) | 15 (16.5%) | 91 (100%) |
| Urban location | 57 (52.3%) | 30 (27.5%) | 22 (20.2%) | 109 (100%) |

Adherence to secondary prophylaxis was also superior in rural and remote settings which may have reduced disease progression (median (IQR) adherence: 45% (28-62) versus 38% (19-53), p=0.02) to severe disease as we highlight in table 2.

There was a higher proportion of non-Indigenous patients living in urban locations 53/342 (15.5%) versus 11/344 (3.2%). These non-Indigenous patients living in urban locations were significantly older (median (IQR) age: 60 (25-75) years) than the Indigenous patients living in rural and remote locations (median (IQR) age: 34 (24-49) years). These urban non-Indigenous patients were more likely to have severe RHD versus the Indigenous patients living in rural and remote locations (20/53 (37.7%) versus 70/333 (21.0%), p =0.008). This is likely related to the fact that - as they were older - more time had elapsed for their RHD to evolve to severe disease.

Historically, there is a stronger relationship between the clinicians that provide outreach services (which commenced in the mid 1990’s) and the RHD control team (given the greater burden of RHD in remote locations). These clinicians may be more inclined to report *any* RHD than their counterparts who have a predominantly urban practice and who may be less inclined to report mild – and clinically less significant – disease. Finally, it may also be the case that patients with severe valvular heart disease were more likely to move to - or stay resident in - an urban setting due to concerns about their underlying heart condition, although we did not have sufficient data to examine this hypothesis in this study.
